# Ring Vibrations to Sense Anionic Ibuprofen in Aqueous Solution as Revealed by Resonance Raman

**DOI:** 10.3390/molecules27020442

**Published:** 2022-01-10

**Authors:** Sara Gómez, Natalia Rojas-Valencia, Tommaso Giovannini, Albeiro Restrepo, Chiara Cappelli

**Affiliations:** 1 Classe di Scienze, Scuola Normale Superiore, Piazza dei Cavalieri 7, 56126 Pisa, Italy; tommaso.giovannini@sns.it; 2 Instituto de Química, Universidad de Antioquia UdeA, Calle 70 No. 52-21, Medellin 050010, Colombia; nandrea.rojas@udea.edu.co (N.R.-V.); albeiro.restrepo@udea.edu.co (A.R.)

**Keywords:** resonance Raman, UV-vis, spectroscopy, NBO, ibuprofen

## Abstract

We unravel the potentialities of resonance Raman spectroscopy to detect ibuprofen in diluted aqueous solutions. In particular, we exploit a fully polarizable quantum mechanics/molecular mechanics (QM/MM) methodology based on fluctuating charges coupled to molecular dynamics (MD) in order to take into account the dynamical aspects of the solvation phenomenon. Our findings, which are discussed in light of a natural bond orbital (NBO) analysis, reveal that a selective enhancement of the Raman signal due to the normal mode associated with the C–C stretching in the ring, $\nu_{C=C}$, can be achieved by properly tuning the incident wavelength, thus facilitating the recognition of ibuprofen in water samples.

## 1. Introduction

**Figure 1:**
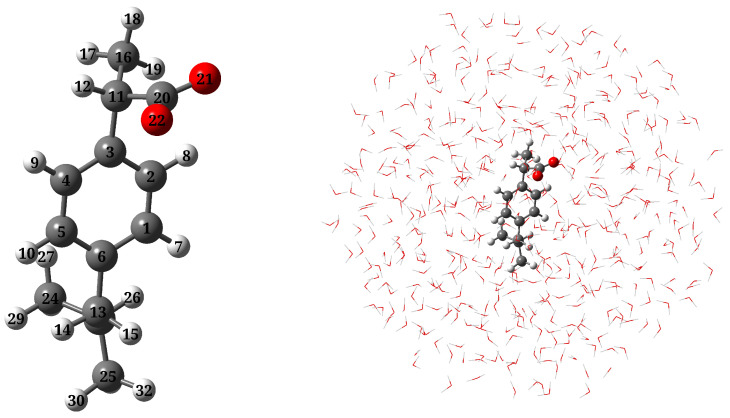
Molecular structure of a-Ibu and atom labeling (**left**); a pictorial view of a-Ibu dissolved in aqueous solution as treated in QM/MM calculations (**right**).

Ibuprofen, a non-steroidal anti-inflammatory agent, has become and remains one of the most consumed medications around the world [1]. Indeed, the worldwide ibuprofen market is projected to increase over the period 2020–2024 [2]. The wide use of ibuprofen is related to its capability to provide therapeutic action for a variety of diseases, Ref. [1] via the inhibition of the COX enzyme [3] during the production of prostaglandins [4]. According to its chemical structure, ibuprofen is classified as an amphiphilic molecule as it presents an aromatic ring doubly substituted with a methyl propyl group and with a propionic acid, the latter constituting the polar part of the drug. The connection between the non-polar and polar groups not only allows the molecule to be soluble in both polar and apolar environments but also gives rise to an enantiomeric carbon from which the *S*-configuration has been regarded as the active species (see Figure 1) [5].

The study of ibuprofen in aqueous solutions is a hot research topic from biological and environmental points of view [6,7,8]. From the biological perspective, the human body is around 70% water, therefore it is expected that water⋯ibuprofen interactions will play an important role in the drug behavior and hence in its therapeutic action [9,10]. On the other hand, ibuprofen is of environmental importance because it is consumed in large quantities and it has been reported as a pollutant that must be removed from residual waters (especially from healthcare and wastewater treatment facilities) [8,11,12,13]. In both cases, the identification of ibuprofen in the aquatic bodies is quite relevant taking into account its protonation state since ibuprofen undergoes structural transitions at acidic or basic pH, and its properties highly depend on it [14,15]. In this regard, ibuprofen as well as, to a greater extent, the anionic ibuprofen (a-Ibu, resulting from deprotonation) have been described as strong chelating agents, acting as monodentate or bidentate ligands through the carboxylate oxygens [16].

Over the years, the removal of ibuprofen from aqueous solutions has drawn great interest. Researchers have suggested several promising strategies as chemical adsorption on different materials, Ref. [17,18,19,20,21] advanced oxidation processes [13,22], coagulation–flocculation [23], photocatalytic degradation [24], among others, focusing on the increase of the removal rate. As for the detection of ibuprofen in water and wastewater, electrochemical techniques [25,26,27,28] seem to be the most investigated, as well as microextraction methodologies [29]. In addition, spectroscopic techniques are well established fundamental tools to identify several analytes in a given aqueous matrix. Among these techniques, UV-vis is one of the most commonly used (by itself or coupled to a separation technique such as HPLC), and calculated UV-vis spectra are also available in the literature to better understand the experimental data [30].

According to the experimental studies carried out by Olaru and Patras [31] and Du et al. [32], it is possible to identify ibuprofen in water by means of UV-vis spectroscopy. However, the spectra from both the protonated and deprotonated drug perfectly match each other, thus suggesting that the excited electronic states involving the apolar part of the drug, which is the only difference between the structures, play a major role than those of its polar component. In order to get better detection and distinction pictures, other spectroscopic techniques have been used. For example, neutral and anionic ibuprofen have been widely studied and characterized by Raman, Raman optical activity (ROA), and IR spectroscopies [33,34,35,36,37,38]. However, due to the very strong IR absorption of water in the 1700–1400 cm${}^{-1}$ region, the use of Raman is recommended [16]. It has been shown that the vibrational modes, especially those of the aromatic ring, change depending on the chemical environment surrounding the molecule, and due to that, they have been regarded as sensors [35]. Notwithstanding, very diluted ibuprofen-containing solutions continue to be a challenge in the detection processes [39,40].

Resonance Raman (RR) has been proposed as a technique that can significantly increase band intensity and resolution compared to conventional Raman, because the incident wavelength is at resonance with molecular electronic transitions [41,42,43,44]. Therefore, RR allows the detection of analytes even at very low concentrations [45,46]. Despite the obvious importance of the detection of ibuprofen in aqueous solution, to the best of our knowledge, there are neither computational nor experimental studies of RR applied to that system, albeit ultraviolet resonance Raman (UVRR) spectra of human serum albumin and its complexes with three types of ligands, including ibuprofen, have been measured at 240 nm [47]. In this work, based on computational simulations of UV-vis, Raman, and resonance Raman spectra of a-Ibu in aqueous environment, we investigate the potentialities of RR to identify this drug in water. The Natural Bond Orbitals (NBO)-aided detailed description of the different electronic transitions that are involved in UV-vis spectra and could be finely tuned in RR, allows us to gather complementary information to understand the underlying causes of intensity enhancements in Raman spectra, which have not been previously explored.

The paper is organized as follows: after describing the details of the computations in the next section, a discussion focused on hydration patterns under the MD and NBO contexts is presented. Then, the results for diverse spectroscopies, UV-vis, Raman and resonance Raman applied to solvated a-Ibu are reported and analyzed. Finally, conclusions are drawn.

## 2. Methods

Ibuprofen can occur in protonated and deprotonated forms depending on the pH of the solution. An initial analysis of protonation with respect to pH was carried out using Marvin Beans version 19.20 [48] and according to the speciation plots, at neutral pH (pH = 7.0) the dominant form of ibuprofen bears a deprotonated carboxylic group. Besides, physicochemical analyses of the quality of wastewater from different effluents have revealed that regardless of the facility, season, level of treatment, sampling point, etc., pH ranges vary from 6 to 10 [49] with an average pH value found to be around 7.5 [50]. With the ibuprofen pKa being 4.91, this compound will primarily exist in the dissociated form in wastewater too. Therefore, we used the a-Ibu in all the following calculations.

The molecular geometry of a-Ibu (see Figure 1, left panel) was optimized by employing the CAM-B3LYP density functional [51] combined with the 6-311++G(*d*, *p*) basis set [52,53]. To ensure that the geometries and calculated properties are actually representative of the overall solvation environment, we carried out a Molecular Dynamics (MD) simulation to sample the solute-solvent phase space [54]. To do that, the optimized structure of a-Ibu was placed in a cubic box with 7390 water molecules, setting the smallest atom⋯wall distance to 1 nm. The MD run was conducted using the GAFF force field [55] with the TIP3P water model [56]. Parameters for a-Ibu were generated from the electrostatic potential and the CM5 charges [57]. Simulations were run in GROMACS 2019.3, [58] for a total production length of 30 ns, and a step-size of 2 fs. Temperature and pressure were maintained at 298.15 K and 1 bar, respectively, using a modified Berendsen thermostat [59] and Parrinello-Rahman barostat [60], with a coupling constant, τ, of 0.1 ps for each. The system was also equilibrated in two ensembles, NVT and NPT, of 1 ns runs, with solvent and solute in separate temperature coupling groups. At the end of the production stage, we sampled 200 uncorrelated configurations at intervals of 10 ps, discarding the first 10 ns. In the chosen frames, the closest water molecules within a radius of 14 Å of a-Ibu were included in a sphere-shaped cut, as depicted in the right panel of Figure 1. Geometric clustering was conducted to identify similar conformations sampled during the MD run. To this end, the *gromos* clustering method from Ref. [61] with a cutoff of 0.13 nm (the average RMSD) was used. This cluster analysis identified 7 representative structures of a-Ibu, illustrated in Figure S1 in the Supplementary Material (SM). Their corresponding occurrences along the MD trajectory and the size of the cluster are plotted in Figure S2 in the SM.

To get insight into solute–solvent hydrogen bonding interactions, we analyzed the MD trajectory by means of radial distribution functions (RDFs), spatial distribution functions (SDFs), and the average number of hydrogen bonds (HBs) between water molecules and the carboxylate group, by using the TRAVIS package [62,63]. Orbital interactions associated to hydrogen bonding were analyzed at the NBO level [64,65,66] and the interaction energies were obtained via second order perturbation corrections to the Fock matrix with the NBO7.0 program [67]. We also borrowed and reoptimized the structures reported in Ref. [7] as being the lowest energy motifs in the quantum mechanics (QM) potential energy surface (PES) for the microsolvation of a-Ibu: W${}_{1}$S${}_{1}$ ($\%x_{i}=$ 94.3), W${}_{1}$S${}_{2}$ ($\%x_{i}=$ 63.4) and W${}_{1}$S${}_{3}$ ($\%x_{i}=$ 44.9), and applied NBO methodologies to quantify and compare the strength of the interactions.

In spectral calculations, solvent effects were described by means of the quantum mechanics/fluctuating charges (QM/FQ) model [54], applied to each of the extracted snapshots. FQ parameters reported in Ref. [68] were exploited. TD-DFT calculations (15 excited states) for each one of the configurations extracted from the MD trajectory were then carried out at the CAM-B3LYP/6-311++G(*d*, *p*) level of theory. It is worth noticing that with this DFT functional and large number of excited states not only intensities and band shapes are accurately modeled, but also the experimental spectra are better reproduced. Reported averaged absorption spectrum was obtained by convoluting peak intensities with Gaussian functions, with a full width at half maximum (FWHM) of 0.5 eV. The orbitals involved in the main transitions were identified and by means of a canonical molecular orbitals (CMO) analysis, we tabulated the leading NBO contributions (bonding, nonbonding, or antibonding) to each canonical Molecular Orbital (MO). Afterwards, the set of geometries extracted from MD simulations was partially optimized [69] and the vibrational calculations were performed on each converged minima. We also recomputed electronic absorption spectra with the optimized snapshots and we obtained essentially the same results as for the non-optimized ones (see Figure S3 in the SM). The Raman scattering spectrum of a-Ibu in aqueous solution was calculated at 532 nm with the QM/FQ protocol [69]. Finally, the QM/FQ methodology described in Ref. [70] was applied to calculate resonance Raman spectra, choosing several incident wavelengths to build the resonance Raman excitation profile (RREP). The Franck Condon Vertical Gradient approximation, successfully used in other works [70,71,72,73], was exploited. For a better visualization, the Raman and RR stick bands were convoluted with a Lorentzian line shape with FWHM of 20 cm${}^{-1}$. Convergence tests indicated that the inclusion of extra (more than 200) uncorrelated snapshots yield unaltered UV-vis, Raman, and RR spectra (see Figures S6–S8 in the SM). All QM calculations were performed using a locally modified version of the Gaussian 16 package [74]. It should be noted that all calculations refer to the *S*-enantiomer.

## 3. Results and Discussion

### 3.1. Hydration Patterns

**Figure 2:**
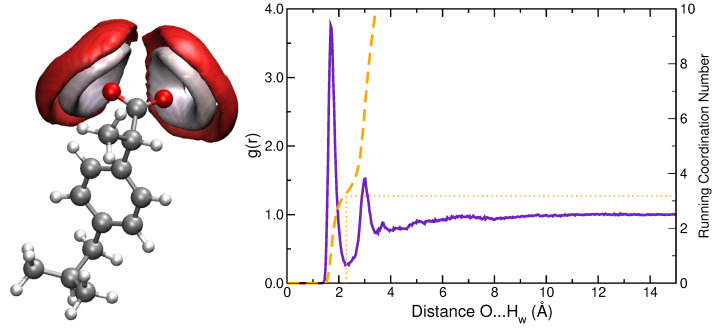
(**Left panel**): Spatial distribution function of water oxygen (red) and hydrogen (white) atoms around a-Ibu. Calculated SDF isodensity values are equal to 70 and 100 nm${}^{-3}$ for hydrogen and oxygen atoms, respectively. (**Right panel**): RDF between ibuprofen oxygens and water hydrogens. The curve is similar for both oxygen atoms in the carboxylate group. Running Coordination Numbers are also included (dashed orange line).

Hydration patterns sampled by MD simulations were analyzed in terms of two descriptors: SDFs and RDFs. Figure 2 shows SDF of solvent atoms (O${}_{w}$ and H${}_{w}$) near oxygen atoms of a-Ibu. Such plots clearly identify the space region occupied by water molecules; the almost symmetrically distributed surfaces indicate that the two oxygen atoms in the CO${}_{2}^{-}$ motif behave in a very similar way. In order to refine the analysis, the RDF between the carboxylate group and water hydrogen atoms was also calculated (Figure 2, right panel). A sharp peak in the *g(r)* can be recognized at 1.7 Å, which integrates for around 3 water molecules located close to each oxygen in the first hydration sphere, whereas a less pronounced peak is present at 3.0 Å, thus denoting a second solvation shell formed by 7 (×2) water molecules. These O⋯H${}_{w}$ distances and arrangements of the solvent molecules surrounding the solute are in line with the findings by Zapata-Escobar et al. [7] who reported a detailed study of the microsolvation of a-Ibu and found that water molecules in direct contact with the solute prefer to aggregate around the carboxylate oxygen atoms via cyclic or bridged charge assisted HBs.

**Figure 3:**
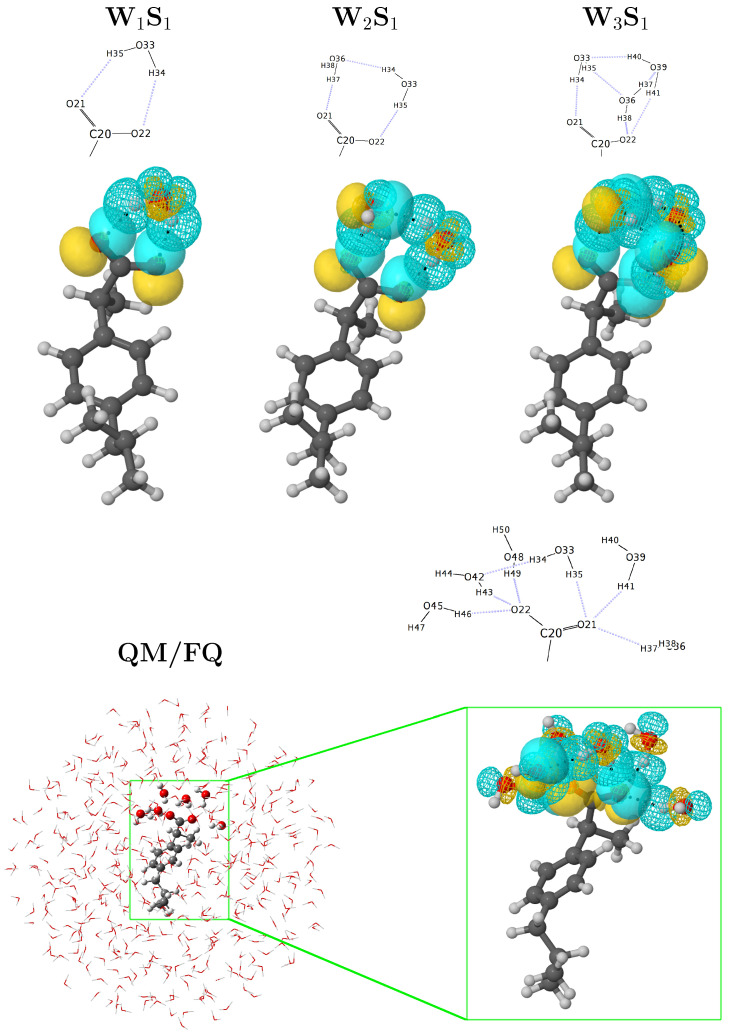
Orbital representation within the NBO picture for the intermolecular interactions in solvated a-Ibu. Structures for W${}_{1}$S${}_{1}$, W${}_{2}$S${}_{1}$, and W${}_{3}$S${}_{1}$ were taken from Ref. [7]. The QM/FQ structure with six explicit waters exhibits the most representative conformation of a-Ibu during the MD run. See also Figure S1 in the SM.

A more comprehensive examination of hydrogen-bonding motifs for the solvated a-Ibu can be done by means of the stabilization energies given by the second order perturbation theory analysis of the Fock matrix on the NBO basis. Such energies allow determining the strength of the donor–acceptor interactions between the solute and the water molecules in its vicinities. Figure 3 compares the intermolecular NBO interactions for anionic ibuprofen and water molecules for the most stable structures reported in the [a-Ibu(H${}_{2}$O)${}_{n}$] *n* = 1, 2, 3 PES [7], with those obtained for a single snapshot (Ibu1 in the SM) extracted from the MD when several water molecules were included in the QM portion. To quantify and compare the relative strengths of the interactions in these molecular clusters, we list the corresponding energies, $E_{{d\to a}^{\left(2\right)}}$, in Table 1.

Even if in the NBO framework there are distinct types of interactions which stabilize the configurations/clusters of a-Ibu in water, the dominant contribution is given by the lone pair → antibonding ($n_{O}\to \sigma_{H-O}^{*}$) form. In all cases, the strongest interactions arise from the charge transfer between a lone pair in the carboxylate group and an antibonding $\sigma_{H-O}^{*}$ orbital of a neighboring water molecule, affording stabilization energies up to 6.81, 26.16, 16.38, and 18.82 kcal/mol when 1, 2, 3 and 6 water molecules (plus the rest of the FQ layer) surround the solute. For clusters with more than one water molecule, the two oxygen atoms are not exactly equivalent and water⋯water interactions are also possible but in general, the $n_{O}\to \sigma_{H-O}^{*}$ overlap leads to small orbital interaction energies, though they are comparable or stronger than the HB in the reference water dimer [75,76] due to the formal charge in a-Ibu. Note that, as predicted by the RDFs, for the configuration considered for this analysis, there are three water molecules located around each oxygen in the a-Ibu. In addition, the $E_{d\to a}^{\left(2\right)}$ energy values, calculated for the interactions in that snapshot, are in the same ranges as those found in a-Ibu/water clusters coming from exhaustive explorations of the PESs, reinforcing the fact that randomly choosing configurations from classical mechanics simulations are reliable sources to gain deep insight about inter-fragment bonding, a strategy that has been recently used in several works [10,77,78,79,80].

### 3.2. UV-Vis Spectrum

**Figure 4:**
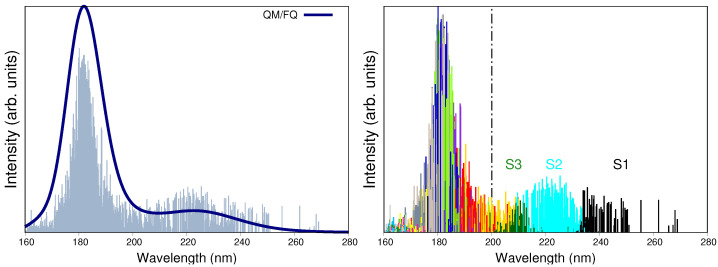
(**Left panel**): Convoluted QM/FQ UV-vis absorption spectrum of ibuprofen in aqueous solution. Convolution was done with Gaussian functions and using a FWHM of 0.5 eV. (**Right panel**): Distinction of the fifteen excited states converged in the TD-DFT calculations, where each one of them is associated to a different stick color. Labels S1 to S3 indicate the first three excited states. Experimental reports collected from Refs. [24,32,81,82] determine the first maximum to appear at 222 nm. Dashed vertical line indicates that there is no experimental information below 200 nm.

We now move to discuss the comparison between computed and experimental UV-vis spectra. Electronic absorption spectra of a-Ibu are usually measured for the ibuprofen sodium salt in solution. When recorded in the 200–300 nm range, the spectrum is characterized by an intense peak with a maximum absorption wavelength ($\lambda_{max}$) of 222 nm, associated with a $\pi \to \pi^{*}$ transition [24,32,81,82]. Other authors claim the main absorption bands to be located at 222 nm and 190 nm, with the latter value being a rough estimation because below 200 nm the spectral data is not conclusive [24]. The QM/FQ calculated absorption spectrum for a-Ibu in aqueous solution is shown in Figure 4, left panel, and the position of absorption maxima (222 nm and 182 nm) in the analyzed interval give a first indication of the good agreement between calculated and experimental data, thus also validating the level of theory here employed. Interestingly, the destruction of the drug by photocatalytic degradation is followed by the disappearance of the band corresponding to ibuprofen at 222 nm [24].

**Figure 5:**
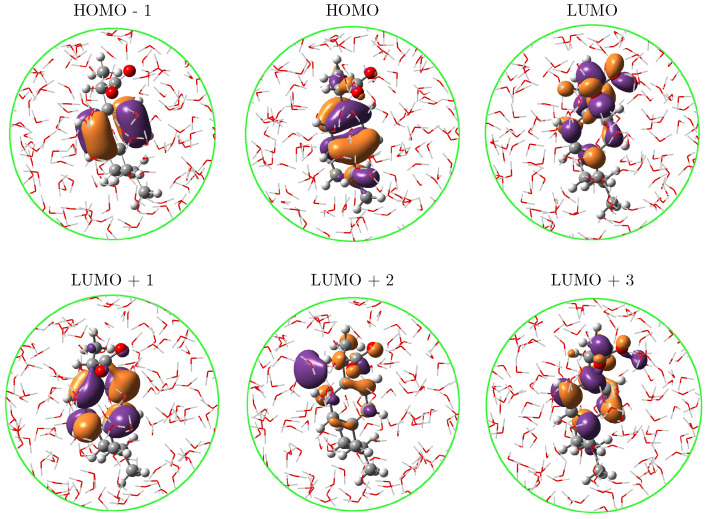
Molecular orbitals for the lowest three excited states of a-Ibu in aqueous solution. See Table 2 for their contributions to the transitions.

As can be seen in Figure 4, left panel, the fact of using a robust sampling methodology that includes several snapshots from the MD brings in a natural broadening in the UV-vis spectra, otherwise lost or arisen from a mere convolution if just a few configurations/conformations are taken into account as it is done in cluster-like approaches or implicit solvation [54]. On the right panel of Figure 4, the stick-like spectrum of a-Ibu was separated by colors according to the excited state the sticks described, in such a way that it is possible to distinguish the excited states mainly contributing to each band. It is clear from such a qualitative separation that $S_{1}$, $S_{2}$ and $S_{3}$ lead to the appearance of the band at higher wavelengths, whereas the second band is due to a combination of all the remaining excitations. In Table 2, the MOs contributions to $S_{1}$–$S_{3}$ transitions are listed. For instance, $S_{1}$ (black sticks in Figure 4) is predominantly a HOMO → LUMO + 1 excitation, whereas $S_{2}$ includes the HOMO as well but the receptor of the charge transfer is the LUMO. The involved MOs are depicted in Figure 5 for the most representative snapshot in light of the clustering method [61].

The NBO analysis of CMO reported in Table 2 allows to further decompose the MOs in NBOs and assign the nature of the transitions. By looking at the values in the linear combinations for the MO already mentioned, it turns out that though the occupied orbitals involved in the transitions might look somewhat different, the NBOs forming them belong to the π aromatic ring in the molecule. Visual inspection of the LUMO orbital in Figure 5 reveals that the carboxylate group plays an important role in that orbital and the CMO decomposition confirms that hypothesis by attributing it primarily to the antibonding $\pi_{C=O}^{*}$ orbital. Gathering all this information, the band at 222 nm in the resonance Raman spectrum of the solvated a-Ibu is a superposition of three excited states, which can be briefly summarized as a redistribution of the electron density from the π-cloud on the ring to either the ring itself or the COO${}^{-}$ groups, in a $\pi_{\mathrm{ring}}$→$\pi_{\mathrm{ring},C=O}^{*}$ charge transfer according to the NBO description.

### 3.3. Raman Spectrum

**Figure 6:**
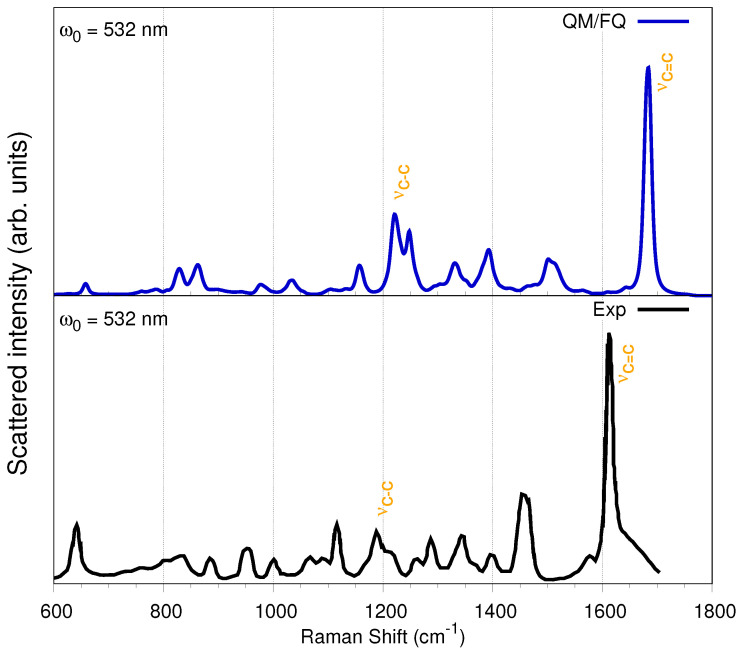
Convoluted QM/FQ (top) and experimental (bottom) Raman spectrum of a-Ibu in aqueous solution. Experimental data taken from Refs. [83,84,85].

The experimental far-from-resonance Raman spectrum of solid IbuNa salt and of its solution in water were reported by Bonora et al. [16] along with the corresponding assignments of the normal modes. Therefore, in what follows, just a summary of the main findings will be discussed. Figure 6 reports the QM/FQ simulated Raman spectrum of a-Ibu in aqueous solution and its experimental counterpart. Overall, there is an outstanding agreement of almost all peak positions and relative intensities.

It has been reported that the most relevant difference between the Raman spectra of the solid undissociated acid form of ibuprofen and a-Ibu in aqueous solution is due to the $\nu_{C=O}$ stretching mode that for the carboxylic group appears at 1647 cm${}^{-1}$ (computed at 1709 cm${}^{-1}$ in Ref. [16]), which is of course not present neither in the diluted IbuNa water solution nor in the solid IbuNa, because in those systems, the asymmetrical $\nu_{as,{\mathrm{COO}}^{-}}$ and symmetrical $\nu_{s,{\mathrm{COO}}^{-}}$ stretching vibrations take place [16].

**Figure 7:**
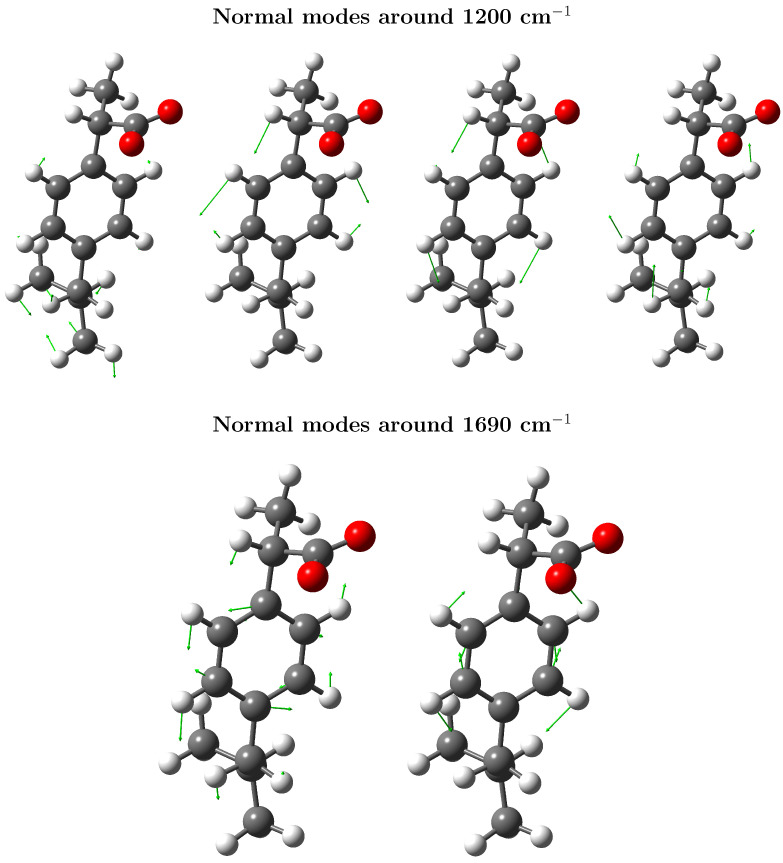
Important vibrational modes giving rise to the most enhanced peaks in resonance Raman. (**Top panel**): normal modes especially enhanced at incident frequencies, $\omega_{0}\approx$ 55,000 cm${}^{-1}$ (182 nm). (**Bottom panel**): normal modes always enhanced according to the RREP, but selectively more enhanced at $\omega_{0}\approx$ 45,000 cm${}^{-1}$ (222 nm).

Concerning the assignments of the bands appearing in Raman spectrum, the most important signals in the 600–1800 cm${}^{-1}$ interval account for an intense peak at 1613 cm${}^{-1}$ (theoretically located at ≈1690 cm${}^{-1}$), attributed to stretching vibrations of the benzene ring ($\nu_{C=C}$). The second most intense peak appears as a doublet with maxima at 1450 and 1455 cm${}^{-1}$ (simulated at 1500 and 1510 cm${}^{-1}$) and can be assigned to the antisymmetric deformations of the methyl groups, the CH${}_{2}$ scissors mode and the $\nu_{s,{\mathrm{COO}}^{-}}$ vibration. Vargek [83] claimed that the splitting could originate from the close proximity of the phenyl ring and the CH${}_{3}$ group in the chiral carbon atom. Another couple of peaks popping up in the spectrum is that at 1288 and 1342 cm${}^{-1}$, which originates from the *out-of-plane* CCCH motion, and the aliphatic $\beta_{\mathrm{HCH}}$ and $\beta_{\mathrm{HCC}}$ bendings. The two Raman peaks at around 800 cm${}^{-1}$ are attributable to torsions $\tau_{\mathrm{HCCC}}$ and $\nu_{\mathrm{CC}}$ stretchings, whereas the peak at 640 cm${}^{-1}$ can be assigned to the phenyl CH *out-of-plane* deformation, though the bending $\beta_{\mathrm{CCC}}$ is also involved. For the upcoming Resonance Raman spectra, it is worth describing the two peaks appearing at 1188 and 1210 cm${}^{-1}$, which are the result of a complex combined $\tau_{\mathrm{HCCC}}$, $\beta_{\mathrm{HCC}}$, $out_{\mathrm{CCCC}}$, and aliphatic $\nu_{\mathrm{CC}}$ vibrations including both the propyl and the isobutyl group, located at the *para-* position from the propanoate (propyl group + carboxylate). CH-bending modes of the benzene ring also contribute to these peaks [47]. The normal modes with frequencies around 1200 and 1690 cm${}^{-1}$ are depicted in Figure 7 for the most representative snapshot selected *via* the *gromos* clustering method. These vibrational modes display large displacements of the C–C bonds.

### 3.4. Resonance Raman

**Figure 8:**
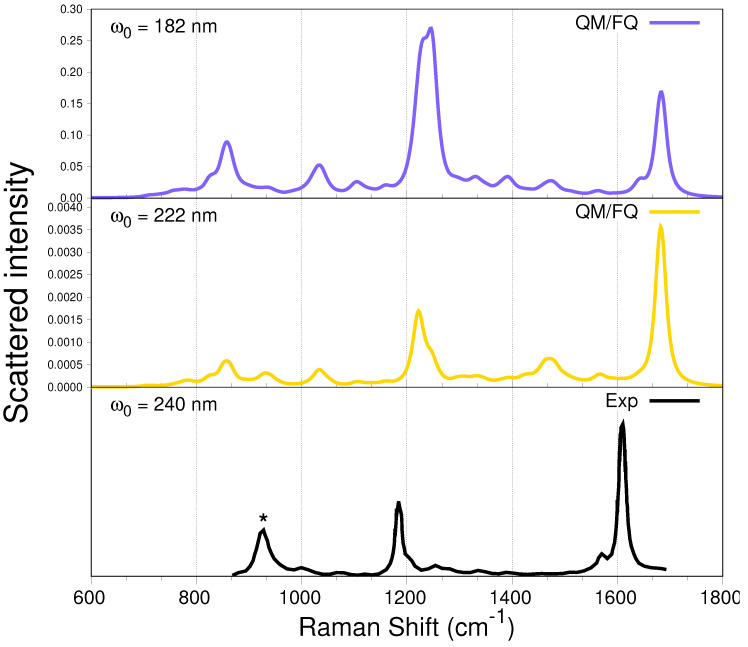
QM/FQ UV-resonance Raman spectra of a-Ibu in aqueous solution, calculated when the two absorption maxima wavelengths are used to irradiate the system ((**top panel**), 182 nm and middle, 222 nm). RR intensities (in cm${}^{2}$mol${}^{-1}$sr${}^{-1}$) were calculated with a damping factor of 200 cm${}^{-1}$ and broadened using Lorentzian functions with FWHM = 20 cm${}^{-1}$. For visualization purposes, the intensity of the highest peak was normalized to 1. In the experimental spectrum [47] (**bottom panel**), the intensity was calibrated with the ClO${}_{4}^{-}$ band marked with ${}^{*}$.

From the simulated UV-vis spectrum of a-Ibu (Figure 4), the lowest electronic absorption with significant oscillator strength was predicted to arise from three excitations, consistent with the appearance of a single, broad band in the aqueous phase absorption spectrum having a maximum (at 222 nm) quite close to what is experimentally observed. Nevertheless, there is another more intense band (at 182 nm) as a result of several transitions with the highest calculated oscillator strengths. The computed RR spectra of a-Ibu when these two incident wavelengths are used to irradiate the sample, are depicted in Figure 8. Spectral profiles for $\omega_{0}$ ranging from 165 to 285 nm are displayed in Figure S4 in the SM. For full clarity, we also computed RR spectra taking into account just the first three excited states, the results are found in the SM (Figure S5).

When compared against the off-resonance Raman spectra in Figure 6, the RR spectra in Figure 8 immediately show evidence of selective resonance enhancement, as is characteristic of RR spectroscopy [86,87,88]. That is particularly evidenced by the slight changes in the position and strong changes in the intensity of some of the peaks described above. Indeed, the major features of the RR spectra of solvated a-Ibu are the enhancements of either the peak around 1200 cm${}^{-1}$ or the peak located at ≈1690 cm${}^{-1}$, both associated mostly with carbon–carbon vibrations in the aliphatic regions and ring, respectively, and to movements of the methyl groups of ibuprofen.

**Figure 9:**
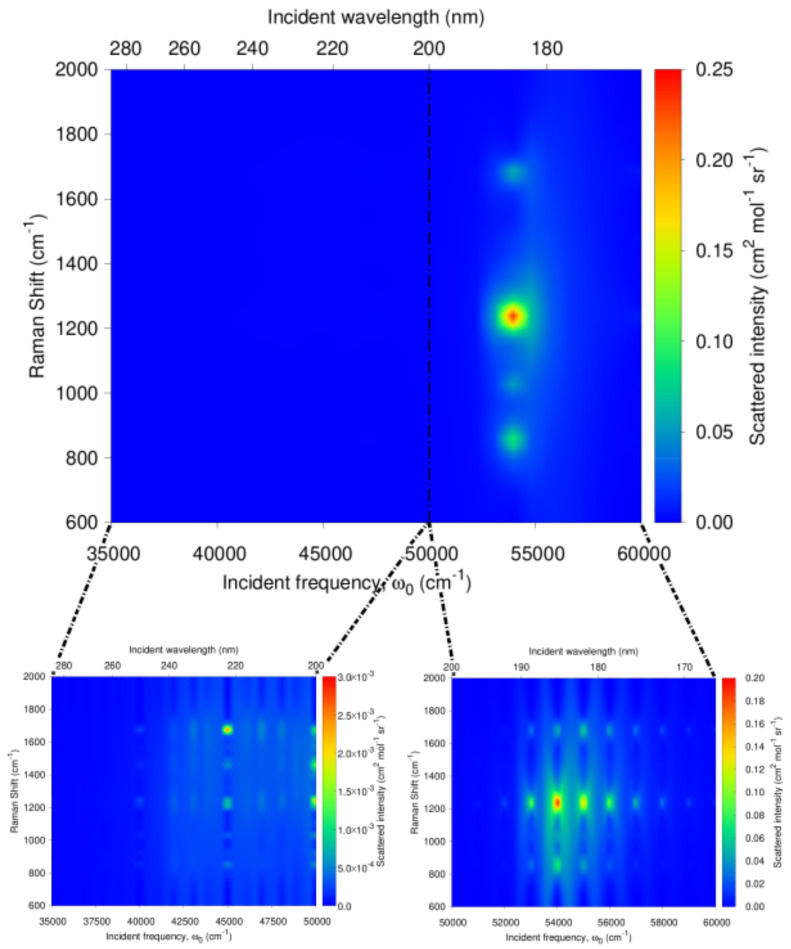
Calculated QM/FQ resonance Raman excitation profile (RREP) of a-Ibu in aqueous solution. Dashed vertical line indicates that there is no experimental information below 200 nm.

Two important findings are noticeable when comparing the two simulated spectra in Figure 8. On the one hand, the RR intensities at 182 nm are about 100 times larger than those at 222 nm (see also Figure 9 below), thus reflecting the relative intensities in the absorption spectra; and on the other hand, there is a selective enhancement of the peak at ≈1200 cm${}^{-1}$ at $\omega_{0}=182$ nm. In fact, at that incident wavelength, this peak exhibits the largest RR intensity, while the peaks located at 1400 cm${}^{-1}$ almost disappeared. Furthermore, notice that at $\omega_{0}=182$ nm, the intensity of the peak at about 800 cm${}^{-1}$ is enhanced.

The only work we found concerning UVRR studies of a-Ibu in aqueous solution, reported measurements with a laser excitation set up to 240 nm, Ref. [47] a near-resonance incident wavelength. For the sake of comparison, that spectrum is included in the bottom panel of Figure 8. The authors pointed out that the most prominent peaks are located at 1613 and 1188 cm${}^{-1}$, assigned to the ring stretching and CH-bending modes of the benzene ring, respectively. Those peaks are characteristic of p-disubstituted benzene derivatives. Our simulations are perfectly consistent with their findings, except perhaps for a small shift in the frequencies.

Going beyond these results at particular excitation wavelengths, the RREP of a-Ibu is shown in Figure 9 as well as a zoom to see in more detail the excitation profile in two specific incident frequency regions: (285–200 nm) and (200–165 nm) under the available experimental information for the absorption spectra. At a first glance, there are at least four peaks (≈800, 1000, 1200 and 1700 cm${}^{-1}$) in the RR spectra expected to increase their intensity when an incident wavelength $\omega_{0}$ = 182 nm is used. Nonetheless, the separation of the regions allows us to recognize that if the threshold of the intensities is lowered, it would be possible to find more enhanced peaks, as seen in the green patches of the RREP maps. While one might argue that this sacrifice in intensity jeopardizes the aim of the detection, for finely tunable devices (like a synchrotron source) used to carry out UVRR experiments, the exploitable range of incident wavelengths is 127–280 nm and moreover, the spectral resolution can be set up for synchrotron radiation-based UVRR experiments in order to have a sufficiently high signal to noise ratio [70,89,90,91].

**Figure 10:**
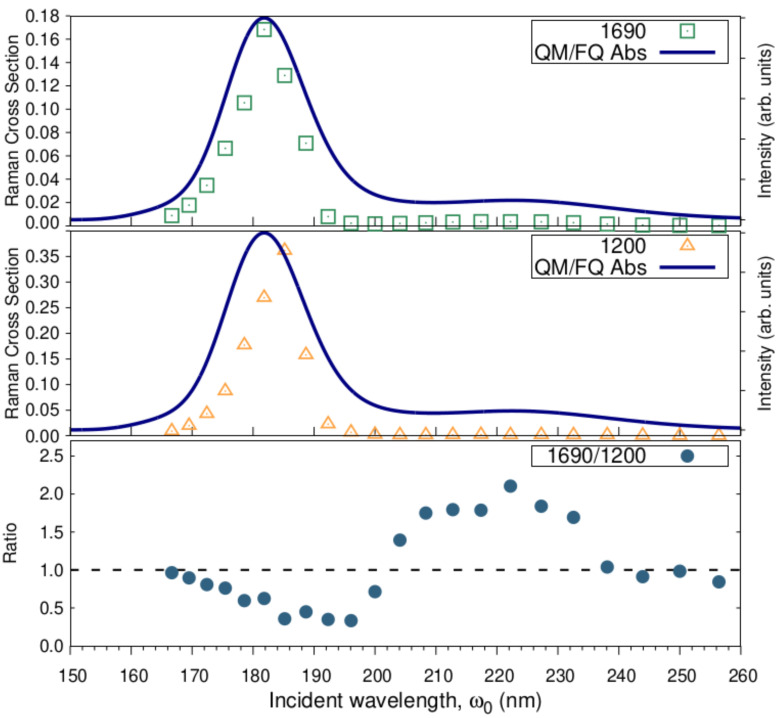
Excitation wavelength dependence of the $\nu_{C=C}$ and $\nu_{C-C}$ bands intensity, and of $\nu_{C=C}$/$\nu_{C-C}$ Raman cross-section ratios. RR intensities (in cm${}^{2}$mol${}^{-1}$sr${}^{-1}$) were calculated with a damping factor of 200 cm${}^{-1}$ and broadened using Lorentzian functions with FWHM = 20 cm${}^{-1}$.

The most enhanced peaks in the RR spectrum of solvated a-Ibu were monitored individually by sectioning the RREP maps. The excitation wavelength dependence of the intensity of the enhanced peaks is shown in Figure 10 along with its Raman cross-section ratios. As expected, the calculated excitation profiles for $\nu_{C=C}$ and $\nu_{C-C}$ reach their maxima at the excitation wavelengths found in the simulated absorption spectra, though the $\nu_{C-C}$ peak shows essentially the same low Raman cross section from 200 to 260 nm.

The selective enhancement of the two signals is also clear from the sinusoidal behavior of the intensity ratios (Figure 10, bottom). Notice that the $\nu_{C=C}$-peak becomes up to two-fold more intense than the $\nu_{C-C}$ peak, for incident wavelengths approaching the second absorption maximum, whereas the ratio equals 0.5 in the vicinities of 182 nm, thus favoring the $\nu_{C-C}$ peak. This fact could be explained by resorting to the information provided by NBO, the analysis of the orbitals involved in the transitions leading to that band, and of course to the normal modes whose signals generate those peaks (see Figure 7). As stated above, the $\pi \to \pi^{*}$ transition predominantly involves orbitals confined to the aromatic ring or the carboxylate group of ibuprofen. What is more, the peak at 1690 comprises normal modes essentially concentrated in the ring, i.e., $\nu_{C=C}$; therefore it is understandable that even at longer wavelengths (200–260 nm) and most likely at 222 nm, an increase in the intensity of this peak is to be observed.

Finally, these results suggest that there are at least two signals to unequivocally trace ibuprofen in aqueous solution through UVRR techniques: (*i*) the aromatic ring $\nu_{C=C}$ stretching mode occurring at 1690 cm${}^{-1}$ and (ii) the aliphatic $\nu_{C-C}$ vibrations emerging at 1200 cm${}^{-1}$, which can be selectively enhanced by choosing the proper incident wavelength. We emphasize here that even if devices with tunable excitation sources are not available for shorter wavelengths, the ring vibrations will appear with an increased intensity when typical 226 nm lasers are used, thus becoming the preferable signals to detect ibuprofen in water.

## 4. Conclusions

In the present study, the potentialities of resonance Raman to detect ibuprofen in water have been investigated by exploiting the mixed quantum–classical polarizable QM/MM scheme based on fluctuating charges to treat in a refined way the solute–solvent interactions, thus obtaining reliable spectral profiles. We additionally have ensured that the anionic ibuprofen⋯water geometries taken for the study are representative of the overall solvated environment by sampling configurations from classical molecular dynamics simulations. As a general rule, hydration patterns indicated that solvent molecules prefer to bind the carboxylate group in a-Ibu, leading to the formation of charge assisted networks of strong hydrogen bonds as confirmed by the NBO analysis.

To validate the application of the QM/FQ + MD methodology for the target system, we have shown that computed vertical absorption energies display a minimal deviation from experimental results, and on top of this, our calculations also capture most of the features of the experimental Raman spectra of a-Ibu in aqueous solution. The validated approach was then used to simulate resonance Raman spectra. The variation of the intensities as a function of $\omega_{0}$ revealed that a couple of peaks in the RR spectrum might be selectively enhanced by properly choosing the incident wavelength. With an NBO-guided assignment of the nature of the electronic transitions, we have related the location of the MOs involved in the excited states with the characteristics of the normal modes whose intensities are enhanced in the RR spectrum. For example, we have outlined that as the $\nu_{C=C}$ normal mode has a large contribution of the C–C stretching in the ring and the $\nu_{C-C}$ does not, therefore the use of $\omega_{0}=$ 222 nm (the maximum absorption for the lowest energy band, which in turn is due to a $\pi_{\mathrm{ring}}$→$\pi_{\mathrm{ring}}^{*}$ transition) would enhance the RR intensity of the $\nu_{C=C}$ normal mode or in general, those normal modes having ring components.

Although the findings in this work provide solid evidence that Resonance Raman can be suggested as a powerful technique to detect ibuprofen in very diluted aqueous solutions, our results would need further experimental verification. Indeed, we are currently in the process of collecting data on that matter, and this will be the subject of a future paper.

## Figures and Tables

**Table 1:** $n_{O}\to \sigma_{H-O}^{*}$ orbital interaction energies in solvated a-Ibu. The largest stabilization energies in kcal/mol are reported in each case. See Figure 3 for atom labeling.

Configuration	Donor (d)	Acceptor (a)	$-E_{d\to a}^{\left(2\right)}$	Type
W${}_{1}$S${}_{1}$	O21	O33-H35	6.77	CO${}_{2}^{-}\cdots$H–O–H
	O22	O33-H34	6.81	CO${}_{2}^{-}\cdots$H–O–H
W${}_{2}$S${}_{1}$	O22	O33-H35	9.37	CO${}_{2}^{-}\cdots$H–O–H
	O21	O36-H37	26.16	CO${}_{2}^{-}\cdots$H–O–H
	O36	O33-H34	7.57	H${}_{2}$O⋯H–O–H
W${}_{3}$S${}_{1}$	O21	O33-H34	16.38	CO${}_{2}^{-}\cdots$H–O–H
	O22	O36-H38	7.84	CO${}_{2}^{-}\cdots$H–O–H
	O22	O39-H41	4.61	CO${}_{2}^{-}\cdots$H–O–H
	O33	O39-H40	9.30	H${}_{2}$O⋯H–O–H
	O36	O33-H35	5.14	H${}_{2}$O⋯H–O–H
	O39	O36-H37	3.21	H${}_{2}$O⋯H–O–H
[a-Ibu(H${}_{2}$O)${}_{6}$]/FQ	O21	O33-H35	18.82	CO${}_{2}^{-}\cdots$H–O–H
	O21	O36-H37	3.19	CO${}_{2}^{-}\cdots$H–O–H
	O21	O39-H41	5.13	CO${}_{2}^{-}\cdots$H–O–H
	O22	O42-H43	10.63	CO${}_{2}^{-}\cdots$H–O–H
	O22	O45-H46	9.60	CO${}_{2}^{-}\cdots$H–O–H
	O22	O48-H49	9.65	CO${}_{2}^{-}\cdots$H–O–H
	O42	O33-H34	3.84	H${}_{2}$O⋯H–O–H

**Table 2:** Decomposition of the excited states mainly contributing to the onset of the band centered at 222 nm in the absorption spectrum of a-Ibu in solution. The decomposition is done based on the canonical molecular orbitals (CMO) and the natural bonding orbitals (NBO).

Excited State	Orbitals Involved		Contribution (%)
	CMO	NBO	
$S_{1}$	HOMO → LUMO + 1	$\pi_{\mathrm{ring}}$→$\pi_{\mathrm{ring}}^{*}$	49.72
	HOMO → LUMO	$\pi_{\mathrm{ring}}$→$\pi_{C=O}^{*}$	29.36
	HOMO - 1 → LUMO	$\pi_{\mathrm{ring}}$→$\pi_{C=O}^{*}$	20.92
$S_{2}$	HOMO → LUMO	$\pi_{\mathrm{ring}}$→$\pi_{C=O}^{*}$	79.80
	HOMO → LUMO + 1	$\pi_{\mathrm{ring}}$→$\pi_{\mathrm{ring}}^{*}$	20.20
$S_{3}$	HOMO - 1 → LUMO + 1	$\pi_{\mathrm{ring}}$→$\pi_{\mathrm{ring}}^{*}$	50.50
	HOMO → LUMO + 3	$\pi_{\mathrm{ring}}$→$\pi_{\mathrm{ring}}^{*},\pi_{C=O}^{*}$	49.50

## Data Availability

Most representative conformers of a-Ibu under clustering method, UV-vis spectra from non-optimized structures, RR spectra at different incident wavelengths, and taking into account just the first three excited states, Convergence tests for UV-vis, Raman and RR spectra.

## Supplementary Materials

The following supporting information is online. Most representative conformers of a-Ibu under clustering method (Figures S1 and S2), UV-vis spectra from non-optimized structures (Figure S3), RR spectra at different incident wavelengths (Figure S4), and taking into account just the first three excited states (Figure S5), Convergence tests for UV-vis, Raman and RR spectra (Figures S6–S8).

## Abbreviations

The following abbreviations are used in this manuscript:

(UV)RR	(Ultra–Violet) Resonance Raman
NBO	Natural Bond Orbitals
FQ	Fluctuating Charges
PES	Potential Energy Surface
MO	Molecular Orbital
CMO	Canonical Molecular Orbital
RREP	Resonance Raman Excitation Profile
QM	Quantum Mechanics
