# Supplementary material for: Ring Vibrations to Sense Anionic Ibuprofen in Aqueous Solution as Revealed by Resonance Raman
Source: Molecules. 2022 Jan 10;27(2):442. doi: 10.3390/molecules27020442 (PMC8780161; doi:10.3390/molecules27020442)
Supplement: Supplementary file 1 [file molecules-27-00442-s001.zip › molecules-1526838-supplementary.pdf]

Ring vibrations to sense anionic Ibuprofen in aqueous solution as revealed by Resonance Raman

Sara Gómez,† Natalia Rojas-Valencia,‡ Tommaso Giovannini,† Albeiro Restrepo,‡ Chiara Cappelli,†

†*Scuola Normale Superiore, Classe di Scienze, Piazza dei Cavalieri 7, 56126, Pisa, Italy*
‡*Instituto de Química, Universidad de Antioquia UdeA, Calle 70 No. 52-21, Medellín, Colombia*

Supplementary Material

Contents

1 Clustering Methodology

2 Additional plots regarding UV-Vis

3 Additional plots regarding RR

4 Convergence plots

1 Clustering Methodology

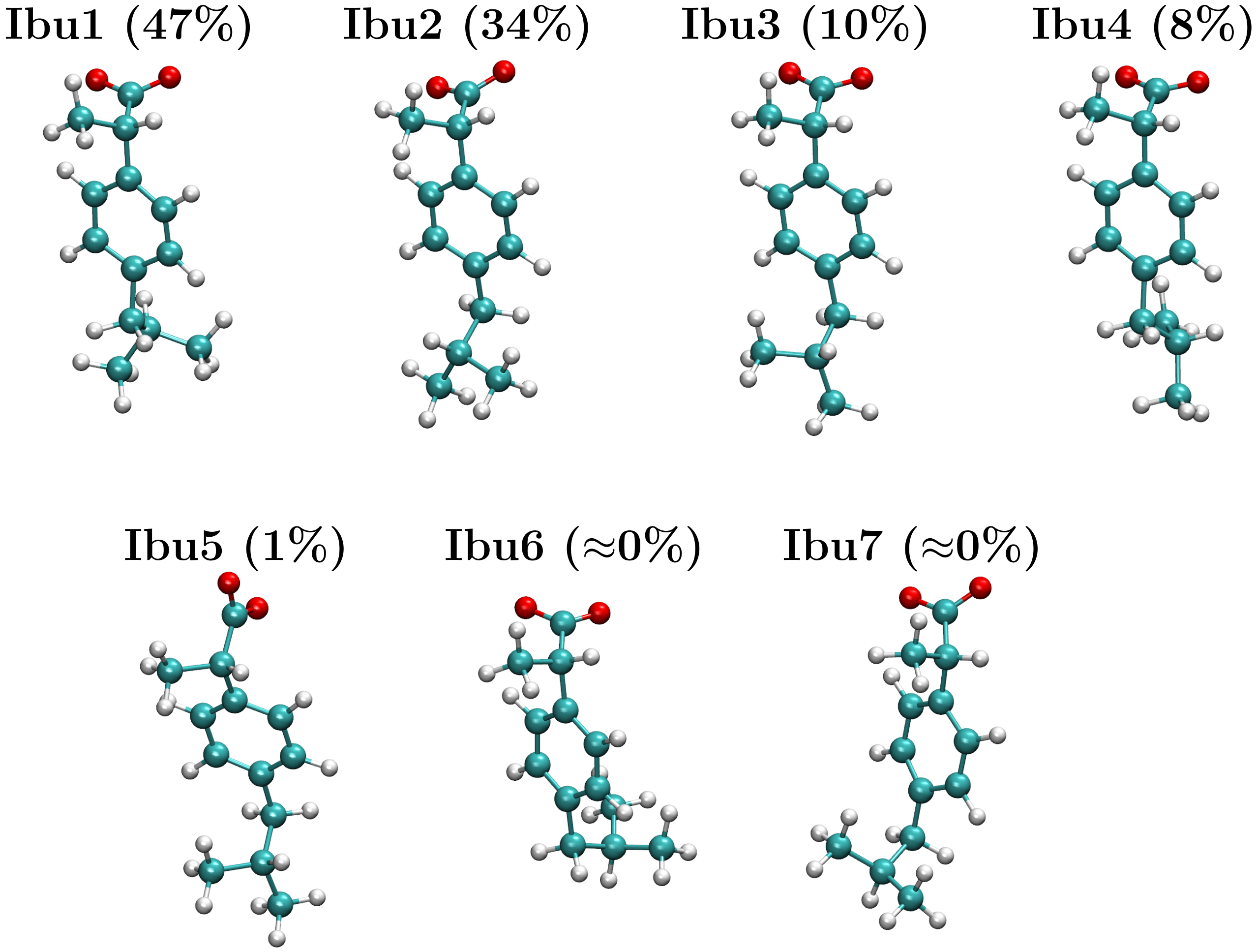

Figure S1: The 7 most populated conformers obtained from a 30 ns MD simulation of a anionic Ibuprofen in aqueous solution at 298 K; clustering performed with a 0.13 nm cut-off with the GROMOS method.

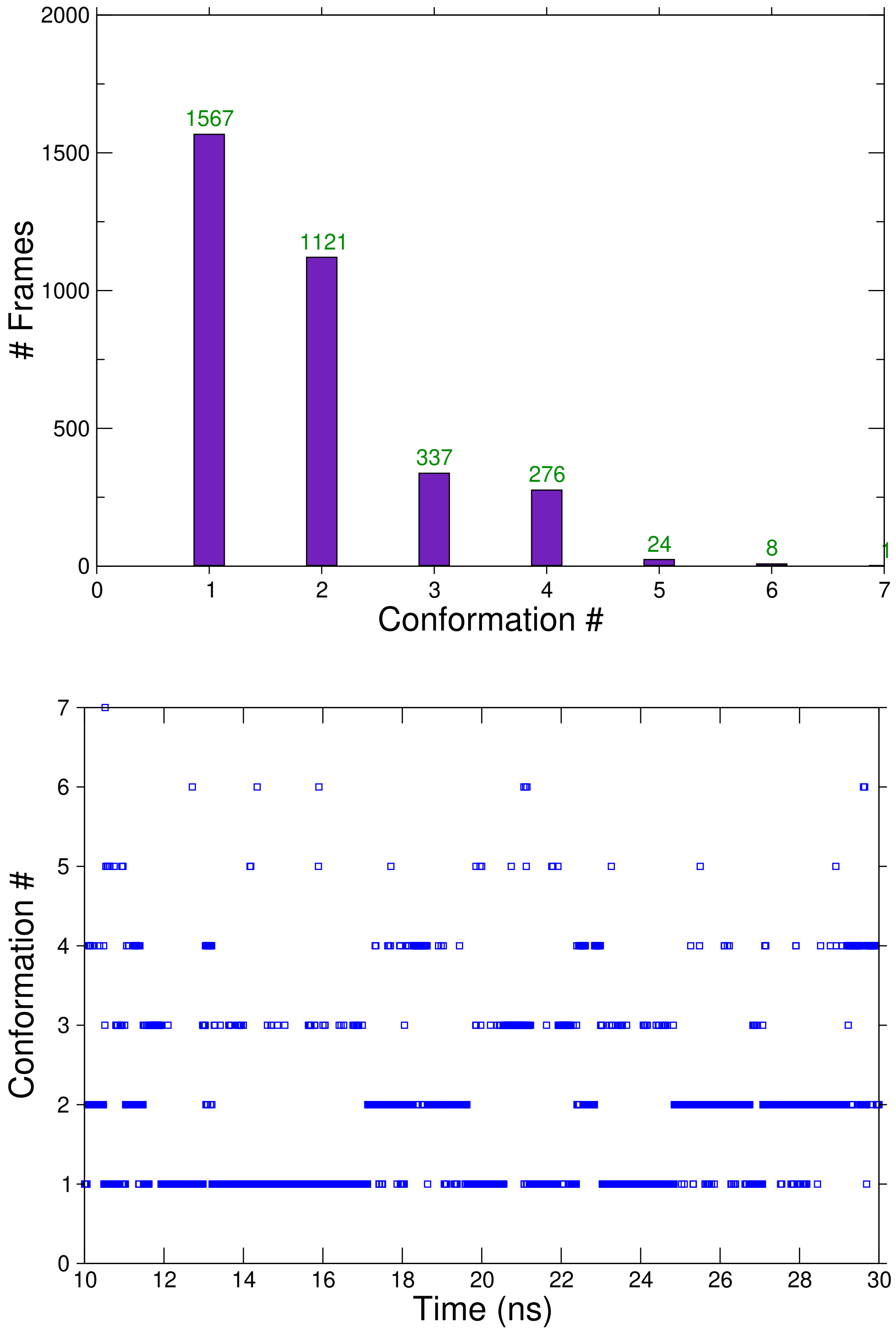

Figure S2: Top panel: Number of frames exhibiting a similar conformation of Ibu^{-}. Bottom panel: the occurrence along the MD simulation of the seven most populated structures. The corresponding conformations are displayed in Figure S1.

2 Additional plots regarding UV-Vis

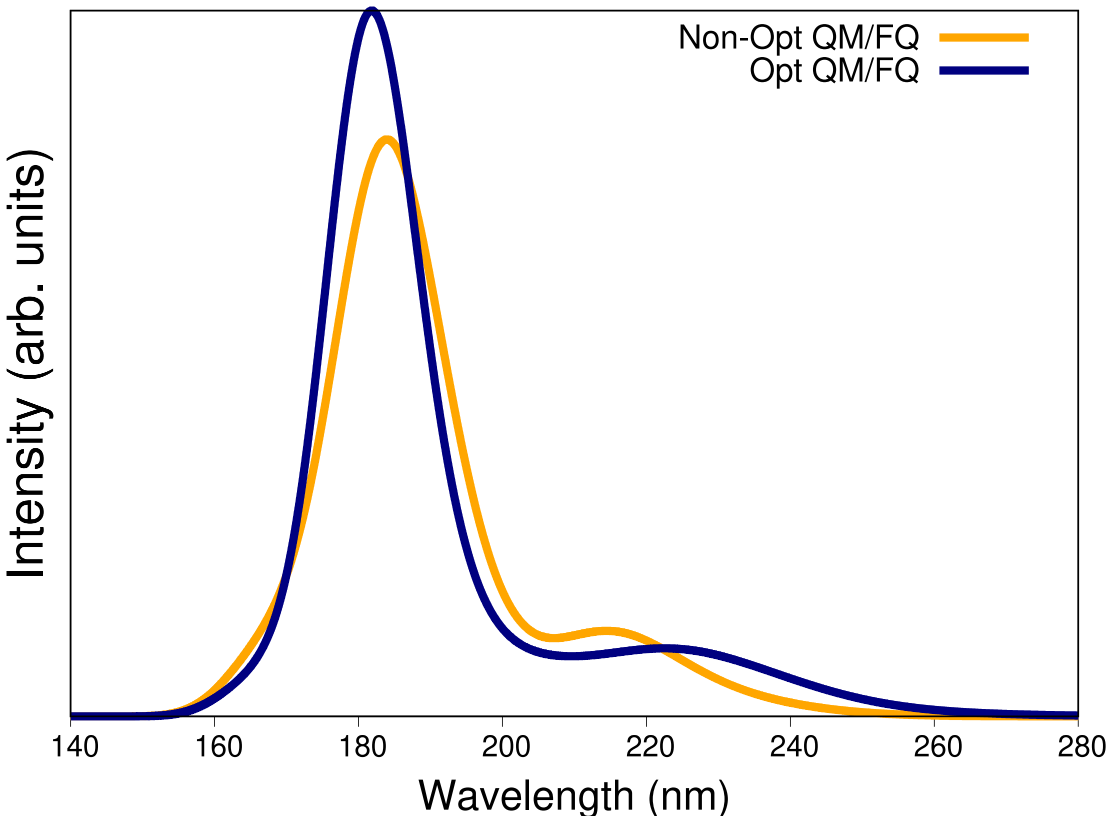

Figure S3: Effect of the optimization on the final UV–Vis spectra of anionic Ibuprofen in water. 200 structures were optimized at the QM/FQ level, with B3LYP/6–311++G(d,p) and then the absorption spectra were calculated in the TD–DFT framework.

3 Additional plots regarding RR

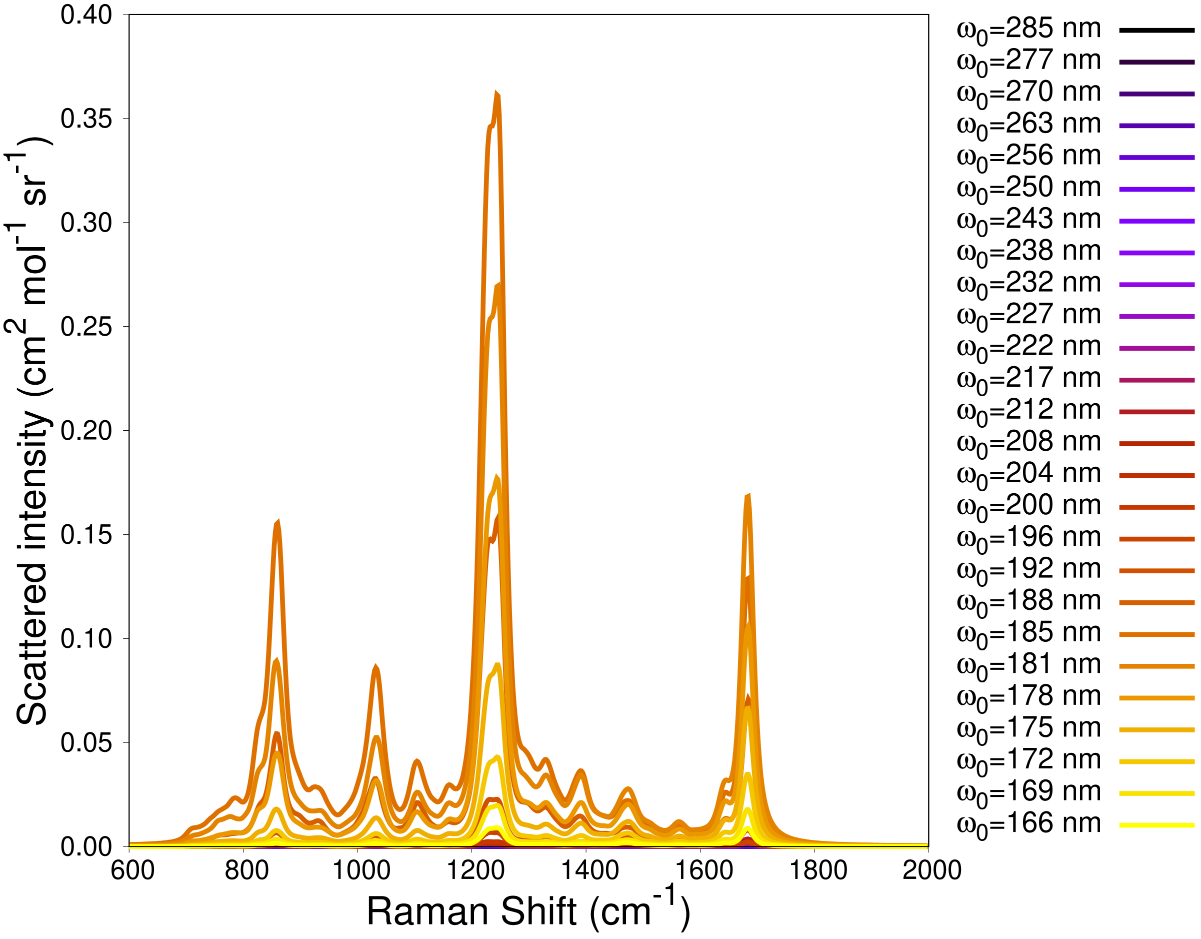

Figure S4: Resonance Raman spectra of anionic Ibuprofen in water, computed at different incident wavelengths.

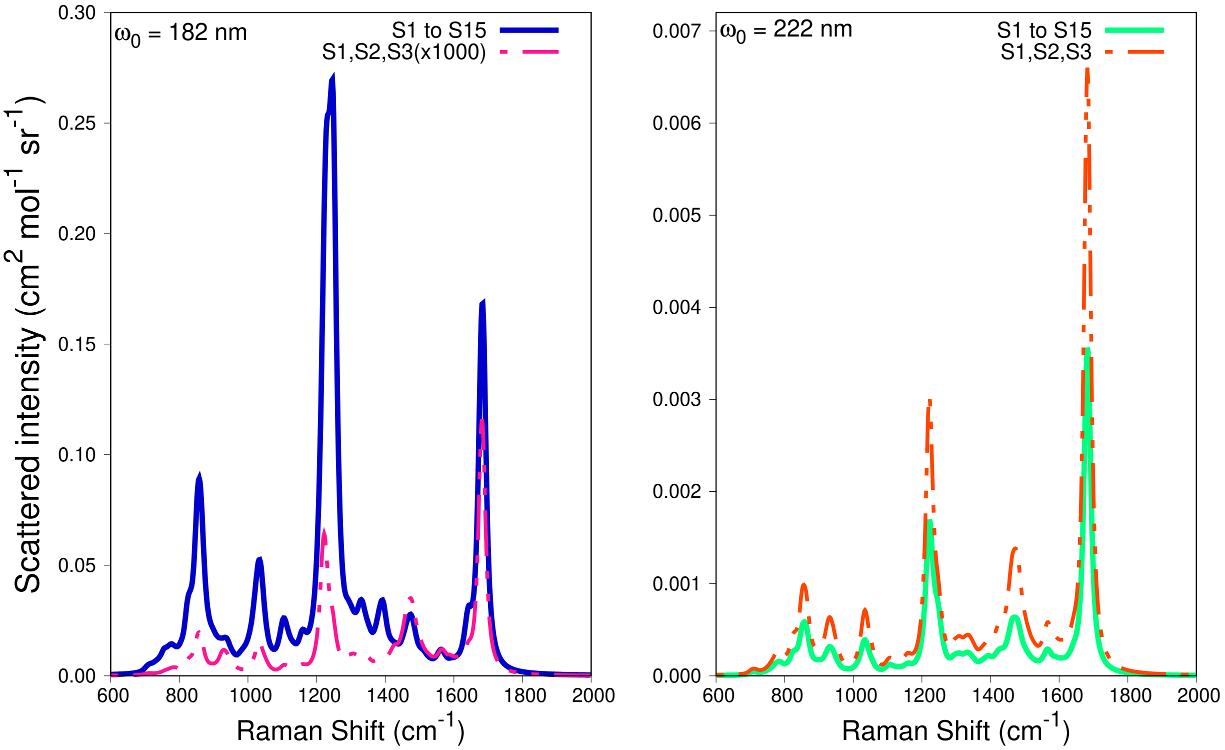

Figure S5: Effect of specific excited states on the selectivity and intensity seen in Resonance Raman spectra of anionic Ibuprofen in aqueous solution.

4 Convergence plots

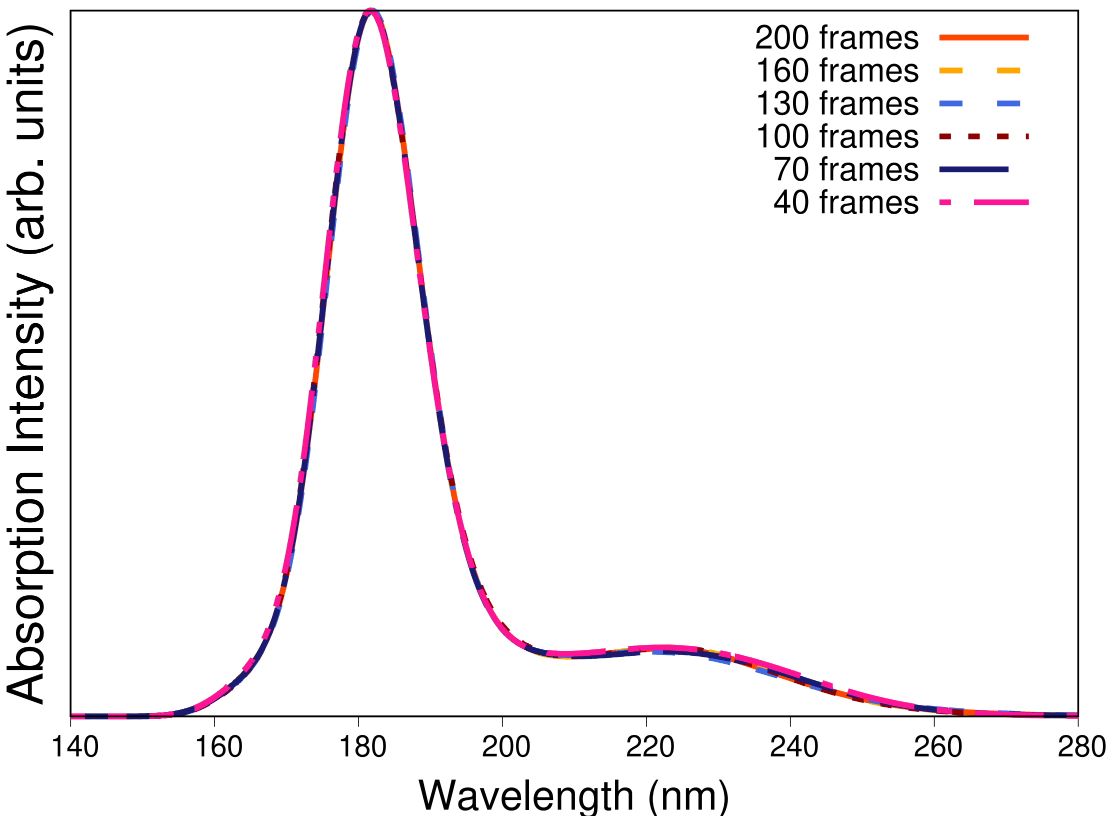

Figure S6: Comparison between the QM/FQ absorption spectra of anionic Ibuprofen in aqueous solution obtained by averaging an increasing number of snapshots.

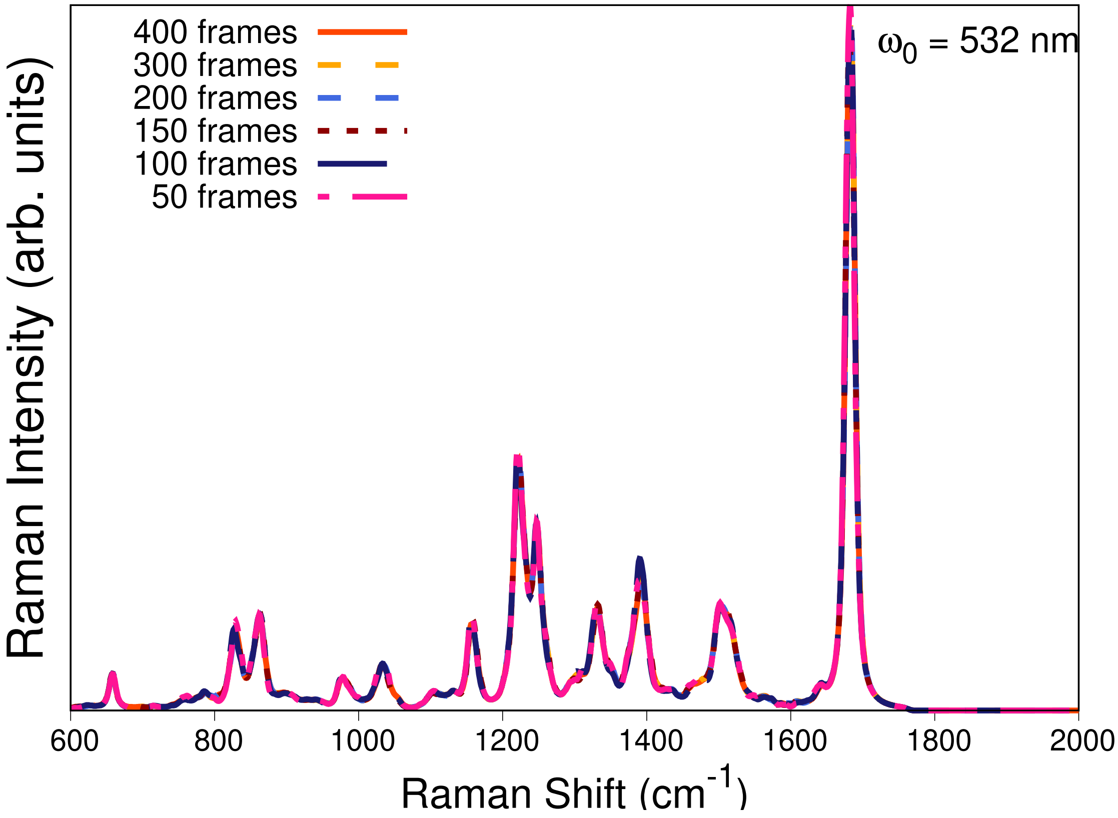

Figure S7: Comparison between the QM/FQ Raman spectra of anionic Ibuprofen in aqueous solution obtained by averaging an increasing number of snapshots.

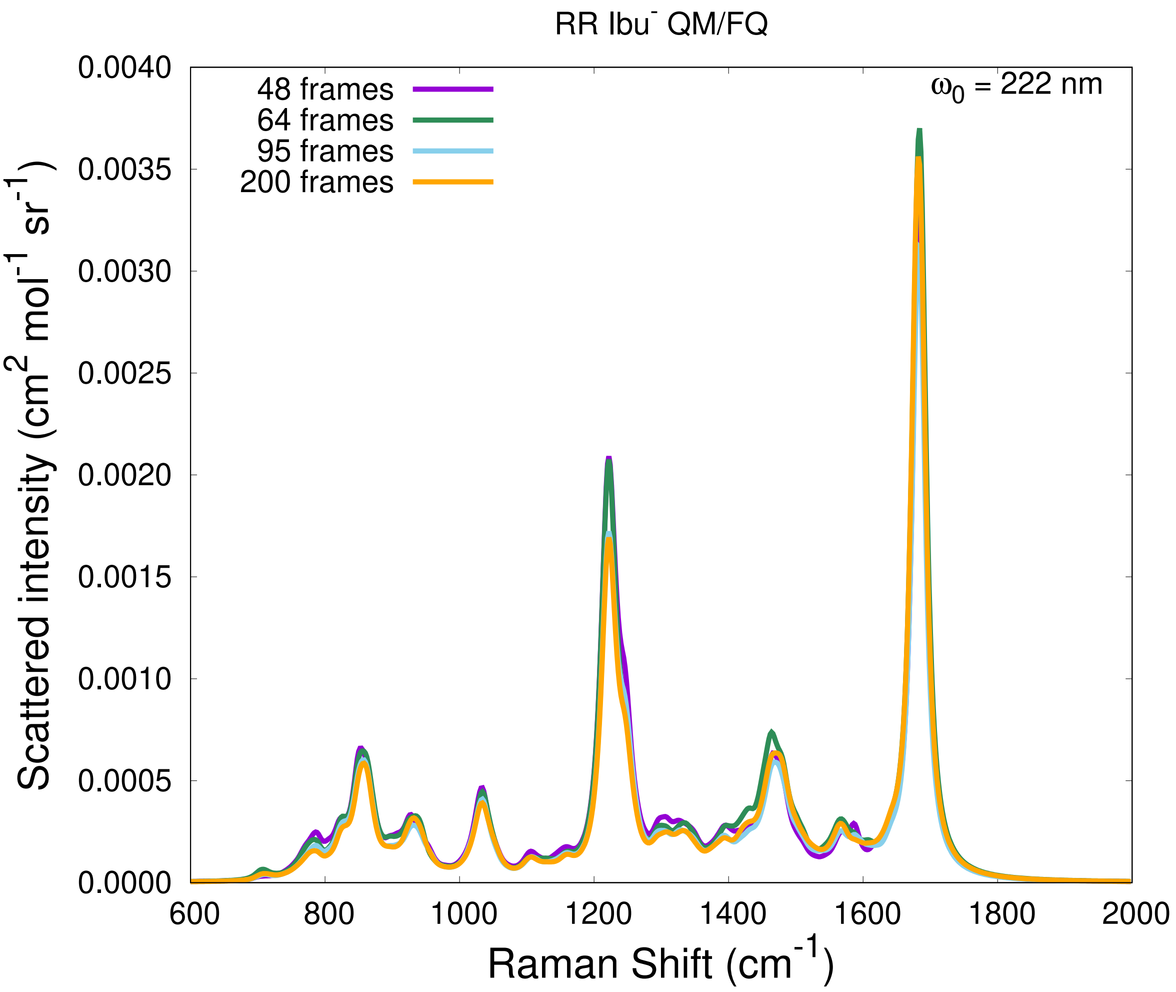

Figure S8: Comparison between the QM/FQ Resonance Raman spectra of anionic Ibuprofen in aqueous solution obtained by averaging an increasing number of snapshots.
